# Dr Dominic Beer DM, FRCPsych

**DOI:** 10.1192/pb.bp.113.046268

**Published:** 2014-04

**Authors:** Stephen Pereira

**Figure:**
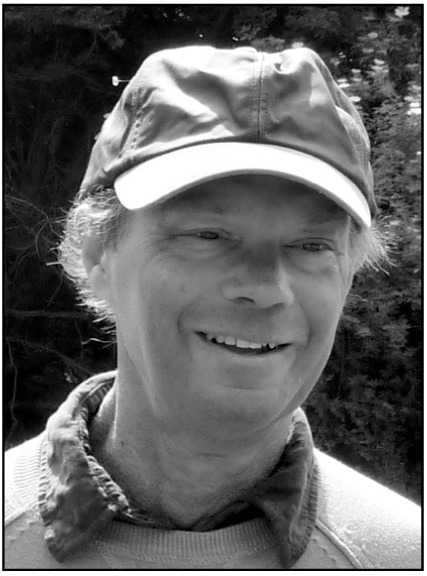


## Formerly Consultant Psychiatrist, Oxleas NHS Foundation Trust

Dominic Beer was one of the pioneers in the reform of psychiatric intensive care units. In the mid-1990s he and his colleagues Carol Paton and myself realised that the quality of care in the locked wards that remained after the opening up of the mental hospitals in the 1950s was extremely variable and sometimes lamentably poor. We carried out a nationwide survey of existing psychiatric intensive care units. This revealed that many units did not have policies relating to admission and discharge; it was often unclear where responsibility for care lay.

We founded the National Association of Intensive Care Psychiatric Units (NAICPU) and in 1996 this held its first national conference. Dominic was elected the first chair of the Association and served from 1997 to 2001. He subsequently served as Treasurer of the Association from 2001 until 2005. The establishment of the Association and the advocacy provided by this group led to considerable improvement in the quality of care delivered to severely disturbed psychiatric patients.

Dominic published over 70 papers, book chapters and books, mainly on psychiatric intensive care but also on a range of other subjects including the history of psychiatry, the psychological impact of abortion and the role of electroconvulsive therapy in treating patients with profound depression. In 2000 he was co-editor of the only international textbook on psychiatric intensive care. A second edition was published in 2008. He was assistant editor of *History of Psychiatry* and refereed papers for a number of other scientific journals.

Dominic was born on 4 November 1956 and educated at Leighton Park School, Reading, where he excelled both academically and in sport. He continued to play cricket well into his adult life and was a member of the Marylebone Cricket Club. In 1975 he went to Wadham College, Oxford, to read modern history and modern languages but changed to read medicine after his second year. He received his psychiatric training at Guy’s Hospital, during which time he took his MD in the history of psychiatry, supported by the Wellcome Foundation. He was appointed to a consultant psychiatrist post at Oxleas NHS Foundation Trust in 1994 where he had responsibility for a 15-bed, low-secure unit at Bexley Hospital.

While he was at Oxford he became a Christian and his faith played a most important part in his life. He was an active member of the Christian Medical Foundation. Although he had a gentle, reflective personality, his immense energy allowed him to carry out his clinical duties most effectively, while at the same time play a most active part in NAICPU, undertake considerable teaching duties and lecture both nationally and internationally on the topic of psychiatric intensive care. He was a great ambassador for British psychiatry abroad. He was Head of Research and Development for Oxleas as well as being Head of Clinical Audit. He also led a very full family and social life. He married Naomi Salter, a general practitioner in east London, and they had four children.

Dominic died peacefully aged 57 years on 19 April 2013 and is survived by his wife and their children.

